# The Interaction between Carbon Monoxide and Hydrogen Sulfide during Chronic Joint Pain in Young Female Mice

**DOI:** 10.3390/antiox11071271

**Published:** 2022-06-27

**Authors:** Gerard Batallé, Xue Bai, Olga Pol

**Affiliations:** 1 Grup de Neurofarmacologia Molecular, Institut d’Investigació Biomèdica Sant Pau (IIB Sant Pau), 08041 Barcelona, Spain; gerard.batalle@autonoma.cat (G.B.); xue.bai@autonoma.cat (X.B.); 2 Grup de Neurofarmacologia Molecular, Institut de Neurociències, Universitat Autònoma de Barcelona, 08193 Barcelona, Spain

**Keywords:** analgesia, allodynia, carbon monoxide, grip strength, hydrogen sulfide, joint pain, oxidative stress

## Abstract

A relationship between carbon monoxide (CO) and hydrogen sulfide (H_2_S) has been described in different pathological conditions, but their interaction in modulating joint pain has not yet been investigated. In young female mice with monosodium acetate-induced joint degeneration and pain, we assessed: (1) the effects of CORM-2 (tricarbonyldichlororuthenium(II)dimer), a CO-releasing molecule, and CoPP (cobalt protoporphyrin IX), an inducer of heme oxygenase 1 (HO-1), administered alone and combined with low doses of two slow-releasing H_2_S donors, DADS (diallyl disulfide) and GYY4137 (morpholin-4-ium 4-methoxyphenyl(morpholino) phosphinodithioate dichloromethane complex) on the mechanical allodynia and loss of grip strength provoked by joint degeneration; (2) the role of Nrf2, NAD(P)H: quinone oxidoreductase 1 (NQO1) and HO-1 in the antinociceptive actions of H_2_S donors; (3) the impact of DADS and GYY4137 treatment on the expression of Nrf2 and several antioxidant proteins in dorsal root ganglia (DRG) and periaqueductal gray matter (PAG). Our data showed that treatment with H_2_S donors inhibited allodynia and functional deficits, while CORM-2 and CoPP only prevented allodynia. The Nrf2 pathway is implicated in the analgesic actions of DADS and GYY4137 during joint degeneration. Moreover, the co-administration of low doses of CORM-2 or CoPP with DADS or GYY4137 produced higher antiallodynic effects and greater recovery of grip strength deficits than those produced by each of these compounds alone. The activation of the antioxidant system caused by H_2_S donors in DRG and/or PAG might explain the enhancement of antinociceptive effects. These data reveal a positive interaction between H_2_S and CO in modulating joint pain in female mice.

## 1. Introduction

The gaseous neurotransmitters carbon monoxide (CO) and hydrogen sulfide (H_2_S) exert multiple biological functions, and there are several similarities between both gases, including: (1) a similar pattern of distribution within the central (CNS) and peripheral nervous systems (PNS); (2) the ability to diffuse freely across intra- and intercellular compartments; (3) both mediate similar physiological functions such as the regulation of cardiovascular, neuronal, respiratory, digestive, and immune systems [1]. In addition, the crosstalk between CO and H_2_S has been described, especially in the gastrointestinal digestive system [2]. For example, Magierowski et al. (2016) [3] demonstrated that H_2_S requires the presence of CO to be able to carry out its protective effects on the gastric mucosa. Other studies also revealed an interaction between both neurotransmitters on the nervous system. Thus, the treatment with electrical acupuncture reduces hypoxic injury because of the improvement of CO levels induced by H_2_S [4]. Moreover, in a rat model of recurrent febrile seizures, the administration of H_2_S donors augmented the plasma levels of CO and the expression of heme oxygenase 1 (HO-1) enzyme in the hippocampus, while the inhibition of H_2_S decreased them [5]. These results suggest a positive interaction between CO and H_2_S in regulating different pathological conditions.

Recent investigations further proved that the acute treatment with slow-releasing H_2_S donors, for example, the natural garlic bioactive element diallyl disulfide (DADS) and the synthetic morpholin-4-ium 4-methoxyphenyl (morpholino) phosphinodithioate dichloromethane complex (GYY4137), both inhibited neuropathic and inflammatory pain in a dose-dependent manner [6,7]. Other findings also show that the administration of the CO-releasing molecule tricarbonyldichlororuthenium(II)dimer (CORM-2) or the HO-1-inducer compound cobalt protoporphyrin IX (CoPP) reduces the inflammatory pain induced by the subplantar injection of complete Freund’s adjuvant [8] and the neuropathic pain associated with type 2 diabetes (db/db mice) [9] or provoked by the administration of vincristine [10], a spared nerve injury [11] and the chronic constriction of the sciatic nerve [12]. Therefore, and considering that joint pain is difficult to treat with the current therapies [13], one of our objectives is to assess the effects of the acute administration of HO-1 inducers and CO or H_2_S donors on the mechanical allodynia and functional disability associated with joint degeneration.

Other works have also showed that most of the anti-inflammatory and antinociceptive effects produced by H_2_S donors during chronic inflammatory and neuropathic pain are mostly mediated by triggering the Nrf2 transcription factor signaling pathway [14,15]. This transcription factor, besides being involved in several protective mechanisms, is also implicated in the maintenance of homeostasis and nociception modulation by activating the transcription of several antioxidant enzymes, such as superoxide dismutase 1 (SOD-1), HO-1, glutathione S-transferase mu 1 (GSTM1), NAD(P)H: quinone oxidoreductase 1 (NQO1), etc. [16,17,18]. Other studies further revealed that most of the anti-inflammatory actions of H_2_S donors were produced by increasing and/or maintaining high protein levels of several antioxidant enzymes in the paw and knee of rodents with chronic inflammation, highlighting the importance of the Nrf2 pathway in the anti-inflammatory properties of H_2_S [7,14]. Nevertheless, the possible participation of Nrf2 signaling in the antiallodynic effects and in the recovery of grip strength induced by H_2_S donors during joint pain has not yet been evaluated.

Finally, and given that most symptoms of pain and functional impairment associated with chronic joint degeneration are more common in women [19], this research was conducted in female mice.

Then, in a monosodium acetate (MIA)-induced model of joint degeneration and pain in female mice [20], we evaluated: (1) the impact of the acute systemic co-treatment of CORM-2 and CoPP with DADS and GYY4137 in the mechanical allodynia and grip strength deficits; (2) the reversion of the effects induced by H_2_S donors with the administration of specific inhibitors of Nrf2, HO-1, and NQO1; and (3) the protein levels of Nrf2, HO-1, NQO1, SOD-1, and GSTM1 in the dorsal root ganglia (DRG) and periaqueductal gray matter (PAG) of animals treated with DADS and GYY4137. Both areas are involved in pain processing, DRG taking part in the transmission of pain from the periphery to the CNS [21] and PAG being a key brainstem nucleus that performs an essential role in the descending modulation of pain [22].

## 2. Materials and Methods

### 2.1. Animals

Female C57BL/6 mice (6–8 weeks old and between 21 and 26 g) obtained from Envigo Laboratories (Barcelona, Spain), which were kept under standard light/dark (12/12 h), temperature (22 °C), and humidity (66%) conditions with free access to food and water, were used. Experiments were performed after 7 days of acclimatization to the housing conditions and conducted between 9 a.m. and 5 p.m. The investigations were performed in conformity with the guidelines of the European Commission’s directive (Directive 2010/63/EU) and Spanish law (RD 53/2013) regulating animal research, and were agreed by the Committee for the Use and Care of Animals of the Autonomous University of Barcelona (protocol number: 9863). Every effort was made to reduce the amount of animals used and their suffering.

Based on the data obtained in a pilot test and accepting a risk of α = 0.05 and β = 0.2 in a two-tailed test, 6 animals per group were needed to recognize as statistically significant the differences between groups in the behavioral experiments.

### 2.2. Induction of Joint Pain

Joint pain was generated by MIA (Sigma–Aldrich, St. Louis, MO, USA) intraarticularly injected under isoflurane anesthesia conditions (i.e., 3% induction and 2.5% maintenance). The right knee joint was shaved and flexed at a 90° angle, and 10 μL of MIA (15 mg/mL) dissolved in saline solution (NaCl 0.9%; SS) was administered. Control animals were injected with the equal amount of SS.

### 2.3. Mechanical Allodynia

Mechanical allodynia was evaluated by measuring the hind paw withdrawal response after stimulation with von Frey filaments. To do this, the animals were put in Plexiglas tubes 9 cm in diameter by 20 cm high placed on top of a wire mesh bottom where filaments of different strengths were introduced (North Coast Medical, Inc., San Jose, CA, USA) in accordance with the up–down paradigm [23]. We started the test with a filament of 0.4 g, and the strength of the following filament was enhanced or diminished in compliance with the animal’s response. A filament of 3.0 g was utilized as a cutoff. We used the Excel program (Microsoft Iberia SRL, Barcelona, Spain), which includes curve fitting of the data, to determine the animal’s threshold of the response. The animals were familiarized for 1 h to the environment prior to starting the test.

### 2.4. Measurement of Grip Strength

The grip strength was measured utilizing a computerized grip strength meter (Model 47200, Ugo Basile, Varese, Italy) according to the method depicted in [24]. To determine the grip strength of the hind legs, the investigator took the animal by the base of its tail, allowing the animal to grab the metal bar of the grip strength meter with both hind legs. The metal bar was coupled to a force transducer that automatically recorded the maximum force of each measurement (g). For each animal, the grip strength of the hind limbs was measured in triplicate. To prevent the mice from grabbing the metal bar with their front legs during the test, mice were first allowed to grab a wire mesh cylinder with their front legs. Baseline grip strength values were recorded for each animal as the mean value of three determinations performed prior to MIA or SS injection. This value was considered 100% grip strength and was utilized as a reference for subsequent determinations.

### 2.5. Western Blot Analysis

Mice injected with MIA or SS were euthanized by cervical dislocation at 29 days post-injection, and the ipsilateral DRG (L3–L5) and PAG were rapidly removed and kept at −80 °C until use. Samples from two animals were pooled into one experimental sample to obtain sufficient protein levels to perform Western blot analysis. We analyzed the protein levels of Nrf2, HO-1, NQO1, SOD-1, and GSTM1 in DRG and PAG. The sonication of tissues was made in cold lysis RIPA Buffer (Sigma–Aldrich, St. Louis, MO, USA). After solubilization for 1 h at 4 °C, crude homogenates were sonicated for 10 s and centrifuged at 700× *g* for 20 min at 4 °C. After that, 60 µg of the supernatant (total protein) was mixed with 4× Laemmli loading buffer and loaded onto 4% stacking/12% separating sodium dodecyl sulfate polyacrylamide gels. Proteins were electrophoretically transferred onto a polyvinylidene fluoride membrane for 120 min and blocked for 75 min with phosphate-buffered saline (PBS; Sigma–Aldrich, St. Louis, MO, USA) containing nonfat dry milk at 5%, Tris-buffered saline with Tween 20 comprising bovine serum albumin at 5% (BSA; Sigma–Aldrich, St. Louis, MO, USA) or nonfat dry milk at 5%, and PBS with Tween 20 containing BSA at 5%. After that, membranes were incubated with specific rabbit primary antibodies, anti Nrf2 (1:150) or HO-1 (1:150) from Abcam, Cambridge (UK), with NQO1 (1:200) or glyceraldehyde-3-phosphate dehydrogenase (GAPDH; 1:5000) from Sigma–Aldrich, St. Louis, MO (USA) and with SOD-1 (1:150) or GSTM1 (1:150) from Novus Biologic, Littleton, CO (USA), overnight at 4 °C. Afterwards, blots were incubated with a horseradish-peroxidase-conjugated anti-rabbit secondary antibody (GE Healthcare, Little Chal-font, UK) for 1 h at room temperature. We used chemiluminescence reagents (ECL kit; GE Healthcare, Buckinghamshire, UK) to detect proteins and the ImageJ program (National Institutes of Health, Bethesda, MD, USA) to perform the densitometric analysis.

### 2.6. Experimental Procedures

First, we investigated the effects produced by various doses of DADS (3–30 mg/kg), GYY4137 (0.4–12 mg/kg), CORM-2 (5–10 mg/kg), and CoPP (0.5–10 mg/kg) or their respective vehicles intraperitoneally administered on the mechanical allodynia and grip strength deficits caused by the joint degeneration, at 29 days after MIA or SS injection (6 mice for group). Mice were tested at 1 h after DADS or GYY4137 injection and at 3 h after CORM-2 or CoPP injection in conformity with other works [6,25,26].

In other groups of experiments, we assessed the possible interaction between H_2_S and CO pathways by evaluating the antiallodynic and the grip strength recovery effects induced by the co-injection of low doses of CORM-2 (5 mg/kg) or CoPP (0.5 mg/kg) with low doses of DADS (3 mg/kg) or GYY4137 (0.4 mg/kg), and the reversion of the antiallodynic and the grip strength recovery effects induced by high doses of DADS (30 mg/kg) or GYY4137 (6 mg/kg) with the administration of 25 mg/kg of ML385 (a specific Nrf2 inhibitor), 10 mg/kg of tin protoporphyrin IX, SnPP (a specific HO-1 inhibitor), or 10 mg/kg of dicoumarol (a specific NQO1 inhibitor).

The doses of ML385, SnPP, and dicoumarol used were in compliance with those used in another study [7], and the mice were tested 1 h after their administration. The doses of DADS, GYY4137, CORM-2, and CoPP that produced the maximal and minimal inhibitory effects were selected as of the dose–response curves completed in this research.

Finally, MIA-injected animals treated with DADS, GYY4137, or vehicle were euthanized by cervical dislocation, and the protein levels of the Nrf2 transcription factor and several antioxidant enzymes (HO-1, NQO1, SOD-1, and GSTM1) in the DRG and PAG were determined by Western blot. In this research, we employed SS-injected mice treated with vehicle as controls (3 samples for every group).

All experiments were performed by researchers blinded to the experimental conditions.

### 2.7. Drugs

DADS, GYY4137, CORM-2, and CoPP were purchased at Sigma–Aldrich (St. Louis, MO, USA). DADS and GYY4137 were dissolved in SS, whereas CORM-2 and CoPP were dissolved in dimethyl sulfoxide (1% in SS). Dicoumarol and ML-385 bought from Eurodiagnostico S.L. (Madrid, Spain) and SnPP purchased from Frontier Scientific (Livchem GmbH & Co., Frankfurt, Germany) were also dissolved in dimethyl sulfoxide (1% in SS). All drugs were administered in a final volume of 10 mL/kg.

All compounds were prepared just before injection. For each group treated with a drug, their control group was treated with equivalent volume of vehicle.

### 2.8. Statistical Analyses

Results are presented as the mean values ± standard error of the mean (SEM). The GraphPad software (version 8.0) was used to perform the statistical analysis.

In the behavioral tests, the analysis of the effects produced by different doses of DADS, GYY4137, CORM-2, CoPP, or vehicle, administered alone and combined, was conducted using one-way ANOVA followed by Student–Newman–Keuls test. The actions of DADS and GYY4137 co-administered with specific Nrf2, HO-1, and NQO1 antagonists were likewise examined with a one-way ANOVA and the Student–Newman–Keuls test.

In our experiments, the antiallodynic effects are shown as the percentage of the maximal possible effect in that test latencies pre- (baseline) and post-drug injection were compared and calculated in agreement with this equation:

Maximal possible effect (%) = [(drug − baseline)/(cut-off − baseline)] × 100

The recovery of grip strength is shown as the percentage of the effect in that the grip strength pre- (baseline) and post-drug injection were compared and computed in agreement to this equation:

Recovery of grip strength (%) = [(drug − baseline)/(baseline)] × 100

Differences in Nrf2, HO-1, NQO1, SOD1, and/or GSTM1 levels in the DRG and PAG were also estimated applying the one-way ANOVA and the subsequent Student–Newman–Keuls test.

A *p* < 0.05 was considered significant.

## 3. Results

### 3.1. The Acute Administration of DADS and GYY4137 Inhibited the Mechanical Allodynia and Grip Strength Deficit Induced by MIA in a Dose-Dependent Manner

**Figure 1:**
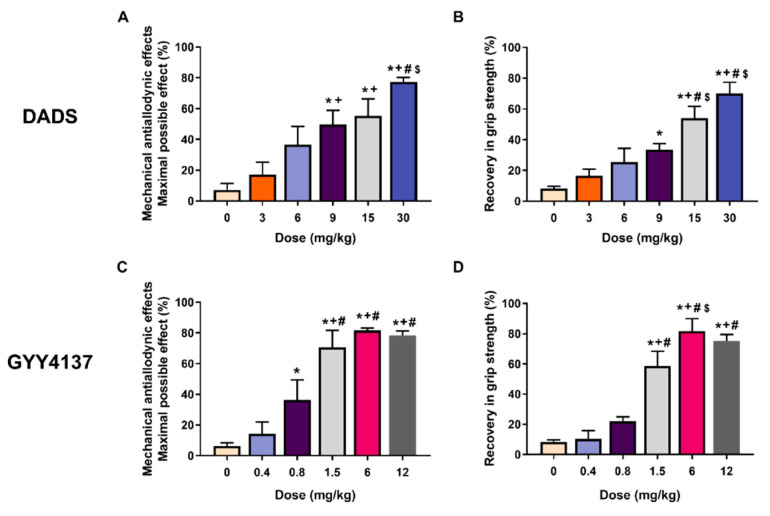
The administration of DADS and GYY4137 reduced the mechanical allodynia and grip strength deficit generated by MIA in a dose-dependent manner. The mechanical antiallodynic effects (**A**,**C**) and the recovery in grip strength (**B**,**D**) induced by different doses of DADS or GYY4137 (mg/kg) are shown. For each test and drug, * denotes significant differences compared to SS (0 mg/kg) treated mice; + compared with the effects of low doses of DADS (3 mg/kg) or GYY4137 (0.4 mg/kg); # compared with the effect of 6 mg/kg of DADS or 0.8 mg/kg of GYY4137 and $ compared with the effects of DADS at 9 mg/kg (*p* < 0.05; one-way ANOVA, followed by the Student–Newman–Keuls test). Data are represented as the mean values of maximal possible effect (%) for the mechanical allodynia and the recovery of grip strength (%) ± SEM; *n* = 6 animals per dose and treatment.

The effects of the acute intraperitoneal administration of diverse doses of DADS (3, 6, 9, 15, and 30 mg/kg) or GYY4137 (0.4, 0.8, 1.5, 6, and 12 mg/kg) on the mechanical allodynia and the loss of grip strength caused by joint degeneration at 29 days after MIA injection were evaluated. Our data showed that acute treatment with DADS dose-dependently reduced mechanical allodynia (Figure 1A) and loss of grip strength (Figure 1B) caused by MIA, achieving the maximum effect in both tests with 30 mg/kg. Treatment with GYY4137 also dose-dependently inhibited the mechanical allodynia (Figure 1C) and grip strength deficit provoked by MIA (Figure 1D) and reached the maximum effect with a dose of 6 mg/kg. In SS-injected animals, both drugs did not make any effect on the allodynia and the grip strength (data not displayed).

### 3.2. Acute Treatment with CORM-2 and CoPP Inhibited the Mechanical Allodynia but Not the Loss of Grip Strength Generated by MIA

The actions of the acute intraperitoneal injection of CORM-2 at 5, 7, and 10 mg/kg or CoPP at 0.5, 2.5, 5, and 10 mg/kg on the tactile allodynia and the loss of grip strength caused by joint degeneration at 29 days after MIA injection were also assessed.

**Figure 2:**
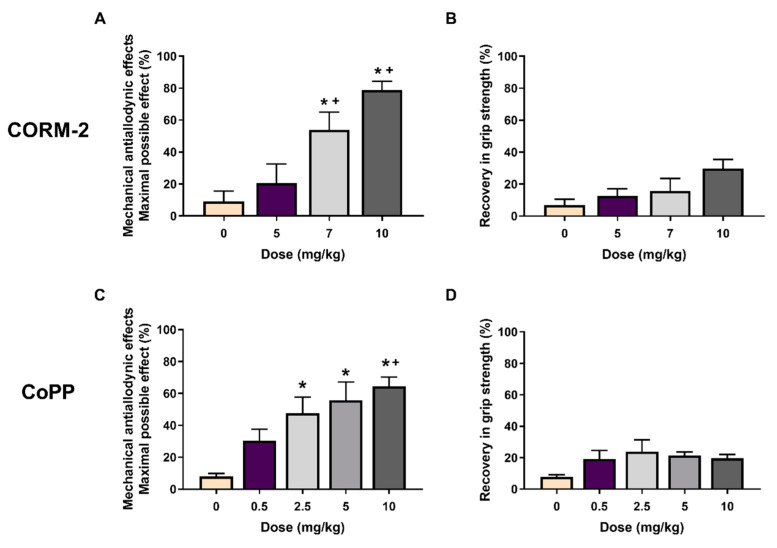
CORM-2 or CoPP treatments inhibited the mechanical allodynia but not the grip strength deficit induced by MIA in a dose-dependent manner. Mechanical antiallodynic (**A**,**C**) and the recovery in grip strength (**B**,**D**) effects induced by different doses of CORM-2 or CoPP (mg/kg) are represented. For each test and treatment, * indicates significant differences vs. vehicle (0 mg/kg)-treated mice and + vs. the effect produced by low doses of CORM-2 (5 mg/kg) or CoPP (0.5 mg/kg) (*p* < 0.05; one-way ANOVA, followed by the Student–Newman–Keuls test). Data are expressed as the mean values of the maximal possible effect (%) for the mechanical allodynia and the recovery in grip strength (%) ± SEM; *n* = 6 animals per dose and treatment.

Our data showed that CORM-2 treatment dose-dependently reduced the mechanical allodynia (Figure 2A), but not the grip strength deficit (Figure 2B), achieving the top effect with a dose of 10 mg/kg. Treatment with CoPP likewise reduced the MIA-induced mechanical allodynia in a dose-dependent way, achieving the greatest effect with 10 mg/kg (Figure 2C) but without effects on the grip strength deficit (Figure 2D).

Both treatments did not show any significant effect on the allodynia and the grip strength of SS-injected animals (data not shown).

### 3.3. The Mechanical Antiallodynic and the Recovery of Grip Strength Effects Induced by the Co-Treatment of Low Doses of CORM-2 or CoPP with DADS or GYY4137 during Joint Pain

**Figure 3:**
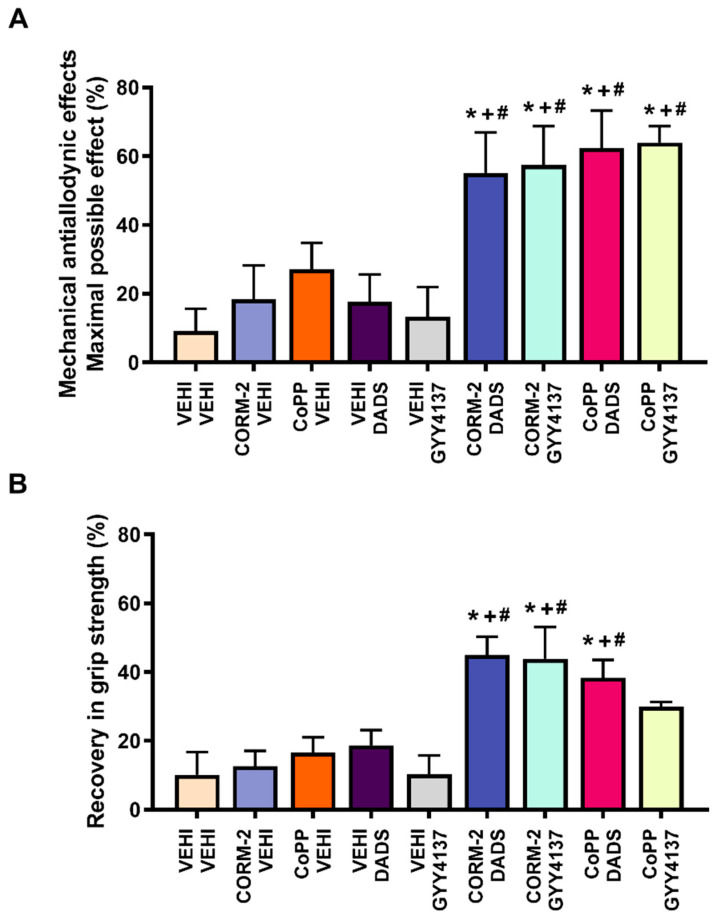
Effects of the co-administration of a CO-releasing molecule and an HO-1 inducer with two H_2_S donors on MIA-induced mechanical allodynia and loss of grip strength. The mechanical antiallodynic effects (**A**) and the recovery of grip strength (**B**) induced by the acute intraperitoneal administration of 5 mg/kg of CORM-2, 0.5 mg/kg of CoPP, 3 mg/kg of DADS, or 0.4 mg/kg of GYY4137 administered alone or combined are displayed. For each test, * denotes significant differences vs. vehicle plus vehicle-treated mice; + vs. CORM-2 or CoPP plus vehicle-treated mice and # vs. vehicle plus DADS- or GYY4137-treated mice (*p* < 0.05; one-way ANOVA, followed by the Student–Newman–Keuls test). Data are expressed as the mean values of the maximal possible effect (%) for the mechanical allodynia and the recovery in grip strength (%) ± SEM; *n* = 6 animals per dose and treatment.

The antiallodynic effects (Figure 3A) and the recovery of grip strength (Figure 3B), induced by low doses of the CO-releaser agent CORM-2 (5 mg/kg) or the HO-1 inducer CoPP (0.5 mg/kg), administered alone and in combination with low doses of DADS (3 mg/kg) or GYY4137 (0.4 mg/kg) were evaluated.

The results showed that the intraperitoneal co-administration of CORM-2 or CoPP with DADS or GYY4137 significantly enhanced the mechanical antiallodynic actions generated by each of them administered alone (*p* < 0.001, one-way ANOVA and Student–Newman–Keuls test; in comparison with their equivalent control groups injected with CORM-2, CoPP or vehicle plus vehicle, and vehicle plus DADS or GYY4137.

Similar findings were detected concerning the recovery of grip strength in which the co-administration of CORM-2 or CoPP with DADS or GYY4137 also displayed significantly greater effects than their corresponding control groups treated with CORM-2 and/or CoPP plus vehicle, as well as versus animals administered with vehicle plus DADS or GYY4137 (*p* < 0.001, one-way ANOVA, Student–Newman–Keuls test). However, although the recovery of grip strength induced by the co-administration of CoPP plus GYY4137 increased, it does not reach statistical significance as compared to those produced by both treatments administered alone.

Finally, neither vehicle injection nor any of the tested combinations exerted any action on allodynia and grip strength in SS-injected animals (data not shown).

### 3.4. Reversal of the Mechanical Antiallodynic Actions and Recovery of Grip Strength Produced by DADS or GYY4137 by Their Co-Treatment with Specific Nrf2, HO-1, and NQO1 Inhibitors

To study the possible contribution of Nrf2, HO-1, and NQO1 in the modulatory effects of H_2_S donors, we assessed the effects produced by high doses of DADS (30 mg/kg) or GYY4137 (6 mg/kg) co-administered with 25 mg/kg of ML-385 (Nrf2 inhibitor) and 10 mg/kg of SnPP (HO-1 inhibitor), or dicoumarol (NQO1 inhibitor) on the mechanical allodynia and the loss of grip strength caused by MIA.

**Figure 4:**
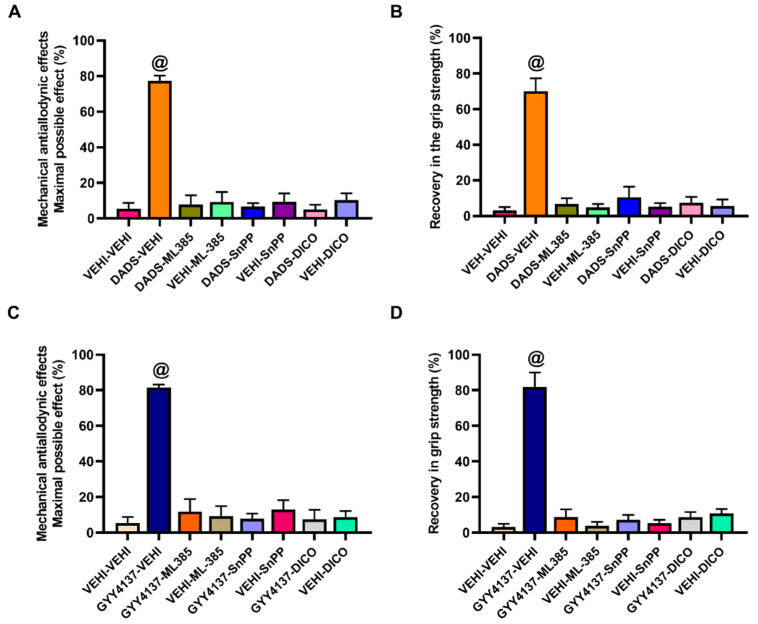
Effects of the co-administration of DADS and GYY4137 with vehicle, ML-385, SnPP or dicoumarol in the mechanical allodynia and loss of grip strength caused by MIA. The antiallodynic (**A**,**C**) and the recovery of grip strength (**B**,**D**) induced by DADS (30 mg/kg) or GYY4137 (6 mg/kg) combined with 25 mg/kg of ML-385 or 10 mg/kg of SnPP or dicoumarol (DICO) are shown. The effects produced by each of these treatments administered alone are also represented. For each treatment and test assessed, @ denotes significant differences vs. the other groups (*p* < 0.05, one-way ANOVA; Student–Newman–Keuls test). Data are expressed as mean values of the maximal possible effect (%) for the mechanical allodynia and the recovery of grip strength (%) ± SEM; *n* = 6 animals per dose and treatment.

The results showed that the antiallodynic effects (Figure 4A,C) and the recovery of grip strength (Figure 4B,D) induced by DADS (Figure 4A,B) or GYY4137 (Figure 4C,D) in MIA-injected animals were completely reversed with the administration of ML-385, SnPP, or dicoumarol (*p* < 0.0001, one-way ANOVA, Student–Newman–Keuls). The administration of ML-385, SnPP, or dicoumarol alone did not alter the mechanical allodynia and grip strength deficit provoked by MIA.

Moreover, treatment with DADS or GYY4137 alone or combined with ML-385, SnPP, or dicoumarol did not exert any effect in SS-injected mice (data not shown).

### 3.5. Effects of DADS or GYY4137 on the Expression of Nrf2, HO-1, NQO1, SOD-1, and GSTM1 on the DRG of MIA-Injected Animals

To evaluate the plausible involvement of the Nrf2 signaling pathway in the inhibitory actions produced by H_2_S donors at the biochemical level, the Nrf2, HO-1, NQO1, SOD-1, and GSTM1 protein levels in the DRG of MIA-injected animals administered with DADS or GYY4137 were assessed.

**Figure 5:**
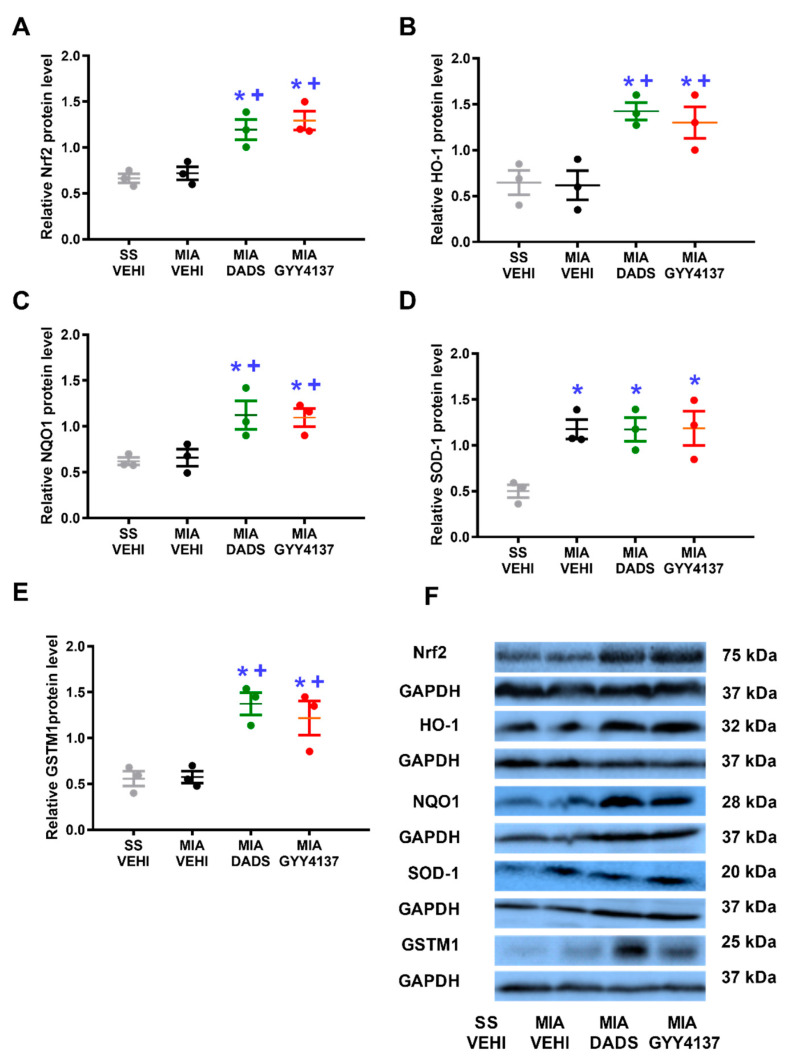
Effects of treatment with DADS and GYY4137 on the Nrf2, HO-1, NQO1, SOD-1, and GSTM1 protein levels in the ipsilateral DRG of MIA-injected mice. The expression of Nrf2 (**A**), HO-1 (**B**), NQO1 (**C**), SOD-1 (**D**), and GSTM1 (**E**) in DRG of MIA-injected animals treated with vehicle (MIA-VEHI), DADS (MIA-DADS) or GYY4137 (MIA-GYY4137) and of SS-injected animals administered with vehicle (SS-VEHI) are represented. All proteins are expressed relative to GAPDH levels. For each protein, * denotes significant differences vs. SS plus vehicle-treated mice and + vs. MIA plus vehicle-treated animals (*p* < 0.05, one-way ANOVA; Student–Newman–Keuls test). Examples of blots for Nrf2, HO-1, NQO1, SOD-1, GSTM1, and GAPDH are shown (**F**). Results are represented as the mean ± SEM (*n* = 3 samples).

Our data showed that MIA injection did not alter the protein levels of Nrf2 (Figure 5A), HO-1 (Figure 5B), NQO1 (Figure 5C), or GSTM1 (Figure 5E), but it significantly increased the expression of SOD-1 (*p* < 0.013, one-way ANOVA vs. SS plus vehicle-injected mice; Figure 5D). Nevertheless, both DADS and GYY4137 increased the protein levels of Nrf2 (*p* < 0.017), HO-1 (*p* < 0.006), NQO1 (*p* < 0.013), and GSTM1 (*p* < 0.002) (one-way ANOVA vs. their respective SS or MIA plus vehicle-treated mice) and retained the upregulation of SOD-1 generated by MIA in the DRG.

### 3.6. Effects of DADS or GYY4137 on the Expression of Nrf2, HO-1, NQO1, SOD-1, and GSTM1 in the PAG of MIA-Injected Animals

To evaluate the effects of DADS and GYY4137 in the expression of Nrf2, HO-1, NQO1, SOD-1, and GSTM1 in CNS of animals with joint pain, the protein levels of these antioxidant enzymes were also assessed in the PAG.

**Figure 6:**
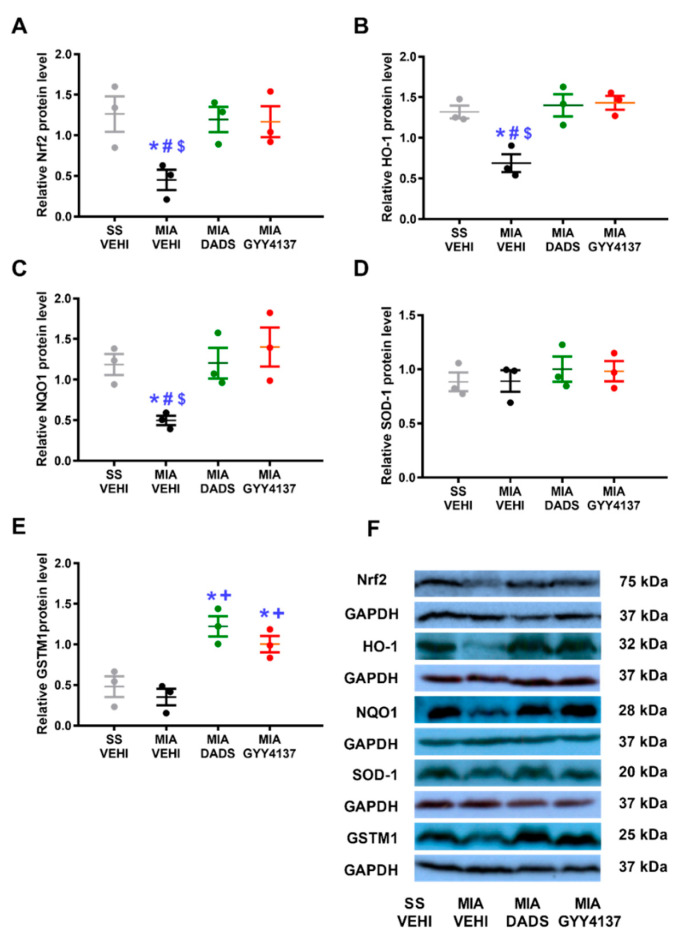
Effects of treatment with DADS and GYY4137 on the Nrf2, HO-1, NQO1, SOD-1, and GSTM1 protein levels in the PAG of MIA-injected mice. The expression of Nrf2 (**A**), HO-1 (**B**), NQO1 (**C**), SOD-1 (**D**), and GSTM1 (**E**) in PAG of MIA-injected animals treated with vehicle (MIA-VEHI), DADS (MIA-DADS) or GYY4137 (MIA-GYY4137) and of SS-injected mice treated with vehicle (SS-VEHI) are represented. All proteins are expressed relative to GAPDH levels. For each protein, * indicates significant differences vs. SS plus vehicle-treated mice; + vs. MIA plus vehicle-treated animals; # vs. MIA plus DADS-treated mice and $ vs. MIA plus GYY4137-injected mice (*p* < 0.05, one-way ANOVA; Student–Newman–Keuls test). Examples of blots for Nrf2, HO-1, NQO1, SOD-1, GSTM1, and GAPDH are shown (**F**). Results are displayed as the mean ± SEM (*n* = 3 samples).

Our data showed that the protein levels of Nrf2 (*p* < 0.036; Figure 6A), HO-1 (*p* < 0.003; Figure 6B), and NQO1 (*p* < 0.024; Figure 6C) diminished in MIA-injected mice (one-way ANOVA; vs. their corresponding SS plus vehicle-treated animals), and both DADS and GYY4137 treatments avoided this downregulation. Furthermore, although MIA injection did not modify the expression of GSTM1 (Figure 6E) in the PAG, both treatments enhanced its expression (*p* < 0.002, one-way ANOVA, as compared with SS- and MIA-injected mice treated with vehicle). Lastly, the expression of SOD-1 (Figure 6D) remained unchanged in the four groups.

## 4. Discussion

This study revealed that the acute administration of an HO-1 inducer, a CO releasing molecule or H_2_S donors inhibited the mechanical allodynia in a dose-dependent manner, but only H_2_S donors inhibited the grip strength deficits caused by joint degeneration. Moreover, the co-administration of low dose of CORM-2 or CoPP with DADS or GYY4137 potentiated the antiallodynic actions as well as the recovery of grip strength produced by each of these compounds. Thus, this suggests a positive interaction between H_2_S and CO on the modulation of joint pain. Our data also revealed the involvement of the Nrf2/HO-1-NQO1 signaling path in the antiallodynic effects and recovery of grip strength induced by both H_2_S donors during joint pain at the pharmacological and biochemical levels.

In accordance with previous studies showing the antinociceptive effects induced by the acute administration of substances capable of releasing H_2_S slowly during neuropathic pain [6], our findings further demonstrated that the acute treatment with DADS and GYY4137 also reduced the mechanical allodynia and functional deficits provoked by joint degeneration in a dose-dependent manner. Furthermore, and considering that the maximum inhibition of mechanical allodynia and grip strength deficit was obtained with 30 mg/kg of DADS, while only 6 mg/kg of GYY4137 was required, these results suggested a greater effectiveness of GYY4137 vs. DADS in terms of inhibition of the mechanical allodynia and grip strength deficits induced by MIA, as previously demonstrated in neuropathic pain [6].

Our data additionally showed that the activation of HO-1/CO signaling with CoPP and CORM-2 treatments induced opposite effects in tactile allodynia and grip strength. That is, both compounds dose-dependently inhibited the mechanical allodynia but did not ameliorate the grip strength deficits provoked by MIA. Thereby, the maximal antiallodynic effects of both compounds were obtained with a dose of 10 mg/kg, but only a 20% recovery of grip strength was observed in all doses tested. In conformity with our results, other works also established the antiallodynic properties of CoPP and CORM-2 during inflammatory and neuropathic pain [25,26], as well as the lack of effects produced by CO inhalation on the grip strength deficits caused by arthritis during the first days of treatment, although a reduction in the loss of grip strength was observed from day 49 of CO inhalation [27]. It is possible that chronic treatment with CORM-2 and CoPP is required for inhibiting the grip strength deficit induced by MIA. In summary, these data showed that under joint pain conditions H_2_S-releasing agents are more effective than HO-1 activators or CO-releasing molecules in inhibiting the grip strength deficits induced by MIA.

The interaction between H_2_S and CO has previously been evaluated in different disorders [4,5], especially in inflammatory processes [3]. In this study, we showed that the co-treatment of CoPP or CORM-2 with two H_2_S donors produced greater antiallodynic effects and the recovery of grip strength than those produced by each of them separately. This revealed a positive interaction between both CO and H_2_S, not only in the antiallodynic effects but in the rescue of the physical disfunction provoked by joint degeneration as well. Thus, this study proposes a new strategy for the management of the allodynia and the loss of grip strength associated with joint degeneration. Moreover, joint pain is difficult to treat with the current therapies [13,28], mainly due to their low efficacy and side effects, especially those affecting the gastrointestinal system [13]. The fact that the co-administration of two gaseous neurotransmitters with important gastroprotective effects [3] produces an increase in their antinociceptive effects is of great relevance for the treatment of joint pain and associated functional disabilities in humans.

To study the possible paths involved in the interaction between these gaseous neurotransmitters, we assessed the role played by the Nrf2 signaling pathway in the effects of H_2_S donors during joint pain. In agreement with other pain models [14], our data further revealed that the antiallodynic effect and recovery of grip strength induced by DADS or GYY4137 were blocked with the administration of specific inhibitors of Nrf2 (ML-385), HO-1 (SnPP), and NQO1 (dicoumarol). This indicates that the activation of the antioxidant system triggered by Nrf2 might be involved in the modulatory role played by both H_2_S donors during joint pain. These findings also suggested that H_2_S effects, including the joint pain modulation, may be dependent on the endogenous CO biosynthesis initiated with the Nrf2/HO-1 pathway activation as occurs with the gastroprotective actions of H_2_S donors [3]. These results agreed with the involvement of the Nrf2/HO-1 system in the painkiller actions of these and other H_2_S donors during inflammatory and neuropathic pain [6,7]. In addition, similar results were obtained in regarding the interaction between CO and other gaseous neurotransmitters such as nitric oxide, where CO needed the presence of nitric oxide to inhibit chronic pain [29].

To evaluate the biochemical interaction between H_2_S and CO, the effects of treatment with DADS or GYY4137 in the expression of the Nrf2 transcription factor and several antioxidant enzymes activated by it (e.g., SOD-1, NQO1, HO-1, and GSTM1) in two areas, one in PNS (DRG) and other in the CNS (PAG), both involved in pain modulation, were evaluated [21,22]. Our data showed that both H_2_S donors increased the Nrf2, HO-1, NQO1, and GSTM1 expression in the DRG, thus revealing that the antioxidative effects of DADS and GYY4137 in joint pain were mainly mediated through triggering the Nrf2 antioxidant signaling pathway activation. These results agree with those obtained in animals with paw inflammation, which also proved the antioxidant capacity of DADS through maintaining elevated levels of HO-1 and NQO1 and normalizing the downregulation of GSTM1 induced by peripheral inflammation in the paw [7]. Our findings further showed a positive interaction between H_2_S and CO in the PNS of animals with joint pain.

It is well known that during chronic pain, several oxidative responses also take place in specific areas of the CNS [30], especially in the PAG, which plays an essential role in modulating descending pain [31,32]. Then, the effects of DADS and GYY4137 systemically administered on the oxidative responses induced by MIA injection in the PAG, as proved with the diminished levels of Nrf2, HO-1, and NQO1 proteins in this brain area, were also evaluated. The antioxidant properties of DADS or GYY4137 in the CNS were demonstrated with the normalization of the downregulation of the Nrf2, HO-1, and NQO1 caused by MIA in the PAG. In addition, both treatments increased the expression of the antioxidant enzyme GSTM1 in this brain area, thus revealing that a positive interaction between H_2_S and CO also occurs in the CNS of animals with joint pain. Our findings were supported by the antioxidant actions caused by garlic consumption in patients with rheumatoid arthritis [33] as well as with the reversion of the oxidative stress produced by treatments with DADS and GYY4137 in the PAG of animals with neuropathic pain [6]. Finally, our results suggested that the activation of the peripheral and central endogenous antioxidant system induced by H_2_S donors may contribute to the increased antiallodynic effects and the recovery of grip strength induced by their co-administration with CORM-2 or CoPP during joint pain.

This study has some limitations such as the fact that it was performed with immature female mice, in a specific model of joint pain, and by the absence of confirmation of the pathology during pain evaluation and its grade during the evaluation of the effects of treatments.

## 5. Conclusions

In summary, this study demonstrated that acute treatment with the H_2_S donors DADS and GYY4137 inhibited the tactile allodynia and the functional deficits provoked by joint degeneration, while CORM-2 and CoPP only inhibited the mechanical allodynia. Moreover, a positive interaction between CO and H_2_S was demonstrated with the enhancement of the antiallodynic effects, and the recovery of grip strength induced by the co-administration of CORM-2 or CoPP with DADS or GYY4137 and with the fact that treatment with H_2_S donors also activated the Nrf2 signaling pathway at the pharmacological and biochemical levels. Therefore, this study suggests that the systemic co-administration of CO and H_2_S activators might be considered as a new strategy for the management of joint pain and the physical disabilities associated.

## Data Availability

Data is contained within the article.
